# Waterproof and Breathable Polyurethane Membranes with Self-Healing and Self-Cleaning Properties: Synergistic Enhancement by Polydimethylsiloxane and Phenolic Carbamate Network and Photocatalytic Effect

**DOI:** 10.3390/polym18070881

**Published:** 2026-04-03

**Authors:** Yuqing He, Xiaohan Yang, Fufen Li, Xiudan Tao, Chenhui Liu, Zhengjun Li

**Affiliations:** 1 Key Laboratory of Leather Chemistry and Engineering of Ministry of Education, Sichuan University, Chengdu 610065, China; 2 College of Biomass Science and Engineering, Sichuan University, Chengdu 610065, China

**Keywords:** waterproof and breathable membrane, waterborne polyurethane, polydimethylsiloxane, tannic acid, self-healing, titanium dioxide photocatalyst

## Abstract

Developing environmentally friendly, multifunctional waterproof and breathable membranes (WBMs) has attracted extensive attention and is of great significance but remains challenging. Herein, an environmentally friendly and multifunctional waterborne polyurethane WBM with self-healing and self-cleaning properties is developed in two steps. Firstly, by using polydimethylsiloxane (PDMS) as a hydrophobicity giver and tannic acid (TA) as a crosslinker, a dual-modified waterborne polyurethane (PTWPU) is prepared, which has high surface hydrophobicity due to the surface enrichment of siloxane segments and self-healing performance from the formation of a dynamic phenolic carbamate network. Secondly, by incorporating titanium dioxide (TiO_2_) photocatalyst nanoparticles to increase internal porosity and establish hydrophilic pathways, a multifunctional waterborne polyurethane WBM (TPTWPU) is developed. This membrane features further enhanced surface hydrophobicity from generated micro-roughness and effective self-cleaning performance, because TA acts as an electron trap to promote the photocatalytic activity of TiO_2_. The TPTWPU membrane shows good hydrophobicity (water contact angle of 115.3°) and satisfactory moisture permeability of 135.0 g/(m^2^·24 h), which is 61.2% higher than unmodified membranes. Furthermore, it exhibits efficient self-healing, with a recovery rate exceeding 80% within 2 h. This feasible strategy will provide guidance for materials design in multifunctional coatings for textiles and leather.

## 1. Introduction

Waterproof and breathable membranes (WBMs) are a class of functional membrane materials that can prevent liquid water from permeating while allowing water vapor to transmit, which have been extensively studied and applied in various fields [1,2]. Commercial WBMs are usually divided into two main categories: hydrophobic microporous membranes and hydrophilic nonporous membranes [3,4]. Among them, a typical hydrophobic microporous membrane, such as polytetrafluoroethylene (PTFE), exhibits excellent water vapor and air transmission, because its internal micropores have sizes between those of water vapor and liquid water droplets, allowing water vapor to pass freely while hindering liquid water infiltration [5,6]. However, fluorine-containing materials possess environmental persistence and bioaccumulation, and their application is restricted due to environmental concerns [7,8]. Regarding hydrophilic nonporous membranes, thermoplastic polyurethane is a representative example. Its water vapor transportation relies solely on an adsorption–diffusion–desorption mechanism driven by internal hydrophilic groups. Consequently, its water vapor permeability is often limited by its dense structure and lack of interconnecting channels [9,10]. In view of the environmental friendliness, versatility and flexibility of waterborne polyurethane, as well as the tailoring of performance, it is urgent to develop a high-level polyurethane WBM by enhancing surface hydrophobicity and establishing more effective internal hydrophilic paths.

For enhancing hydrophobicity, PDMS is often used as an alternative to fluorinated materials because of its low surface energy, biocompatibility, and high thermal stability [11,12]. When PDMS is introduced into polyurethane molecular chains, the incompatibility between nonpolar siloxane segments and polar hard segments causes siloxane to migrate toward the membrane surface. This process forms a low-surface-energy layer that significantly improves the water resistance of the membrane [13,14]. Moreover, the flexible chain segments of polysiloxane can form a microphase separation structure with the hard segments of polyurethane, which will enhance the cohesion through hydrogen bonding and physical entanglement, improving the toughness of membranes [15,16].

To engineer more effective internal hydrophilic paths, a feasible strategy is to introduce hydrophilic functional fillers. This allows for the simultaneous adjustment of internal hydrophilicity and the reasonable construction of micropores. As we know, titanium dioxide (TiO_2_) is a kind of nanoparticle which is easy to prepare and widely used, and its molecular surface contains a large number of hydroxyl groups, showing hydrophilicity [17]. When compounded with waterborne polyurethane, TiO_2_ nanoparticles form hydrophilic paths in the polyurethane matrix. These paths allow water vapor molecules to migrate easily through the adsorption and desorption of hydroxyl groups [18,19]. Additionally, a certain degree of incompatibility leads to the formation of micropores between inorganic nanoparticles and organic polymers, further improving the moisture permeability of the membrane. In addition, it is noteworthy that anatase TiO_2_ nanoparticles have high photocatalytic activity, which can give the membrane good photocatalytic activity and self-cleaning ability after being loaded on the waterborne polyurethane matrix [20,21].

What needs special attention is that with the great progress of society and people’s desire for a comfortable and healthy life, single-function design increasingly fails to meet the evolving demands, so multifunctional materials are urgently needed. For instance, Yi et al. [22] synthesized a waterborne multi-branched sulfonated wood-based polyurethane coating that integrates waterproofing, breathability, antimicrobial activity, and UV protection. Xia et al. [23] fabricated a TPU nanofiber membrane embedded with AgNPs, exhibiting robust antimicrobial performance, washing durability, and waterproof–breathable properties. These studies highlight a clear trend toward next-generation WBMs that integrate diverse functionalities, such as self-cleaning, photocatalytic degradation, and thermal regulation. Such multifunctional materials are essential to meet complex real-world requirements in smart textiles, medical textiles, and outdoor apparel.

Fortunately, we have noticed that tannic acid (TA), which is rich in phenolic hydroxyl groups and derived from biomass resources, can be introduced into polyurethane networks as a natural crosslinking agent to enhance thermal stability and mechanical properties. In particular, the reaction between the phenolic hydroxyl groups of TA and the isocyanate groups leads to the formation of dynamic phenolic carbamate bonds within the polyurethane matrix [24,25,26]. Unlike traditional aliphatic carbamate linkages, phenolic carbamate bonds possess a dissociative dynamic covalent nature. These bonds can undergo reversible dissociation at elevated temperatures, regenerating free isocyanate and phenolic hydroxyl groups, which subsequently recombine upon cooling [27]. Upon physical damage, this dynamic behavior allows the crosslinked network to temporarily de-crosslink, facilitating the diffusion of polymer chains across the crack interface. Subsequently, as the material cools, the dissociated bonds re-form to bridge the gap and restore structural integrity [28]. This process endows the membrane with efficient self-healing performance. Furthermore, our research group also found that TA can promote TiO_2_ to exert its photocatalytic performance and enhance the self-cleaning performance of the membrane.

In this study, we successfully prepared a multifunctional waterborne polyurethane WBM with simultaneous self-healing and self-cleaning properties. This was achieved by using PDMS as a hydrophobicity provider, TA as a crosslinker, and TiO_2_ nanoparticles as a hydrophilic maker. Thanks to the migration and enrichment of nonpolar siloxane chains to the membrane surface and the micro-roughness formed by TiO_2_ nanoparticles on the membrane surface, the membrane was endowed with high hydrophobicity. Benefiting from the hydrophilic channels constructed by TiO_2_ nanoparticles in the polyurethane matrix and the micropores formed by incompatibility with organic polymer chains, the membrane has good water vapor permeability and breathability. Moreover, the membrane possesses self-healing and self-cleaning properties due to the dynamic network of phenolic carbamates and the photocatalytic effect of TiO_2_ nanoparticles. To the best of our knowledge, no similar multifunctional WBMs have been reported previously. This feasible strategy for fabricating environmentally friendly multifunctional waterborne polyurethane WBMs is expected to meet the diverse requirements of potential practical applications.

## 2. Experimental Section

### 2.1. Experimental Materials

Isophorone diisocyanate (IPDI, industrial grade) was purchased from Huaxia Chemical Reagent Co., Ltd. (Chengdu, China), bis(hydroxyethyl)-terminated polydimethylsiloxane (PDMS, Mn = 5000, industrial grade) was purchased from Slokoh Polymer Co., Ltd. (Guangzhou, China), dimethylolpropionic acid (DMPA) and propylene glycol (PPG, Mn = 1000) were purchased from Macklin (Shanghai, China), and 1,4-butanediol (BDO), N, N-dimethylformamide (DMF), dibutyltindilaurate (DBTDL) and 4 Å molecular sieves were obtained from Kelong Chemical Reagent Co., Ltd. (Chengdu, China). Tannic acid (TA, 98%, Mw = 1701.2) was supplied by Shanghai Aladdin Biochemical Technology Co., Ltd. (Shanghai, China). Anatase nano-titanium dioxide (TiO_2_) particles were produced in our laboratory [20]. PDMS and PPG were dehydrated under reduced pressure at 120 °C for 2 h before use. BDO and DMF were dried using 4 Å molecular sieves before use. All of the reagents described above, except IPDI and PDMS, were analytical grade.

### 2.2. Synthesis of Synergistically Modified Waterborne Polyurethane (PTWPU)

**Scheme 1:**
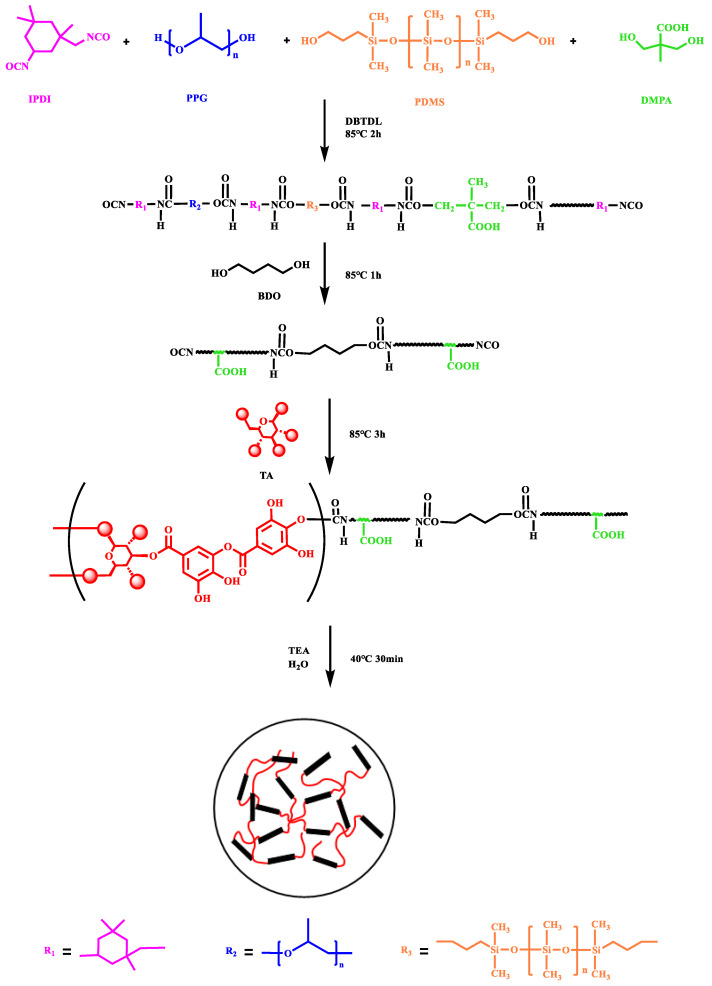

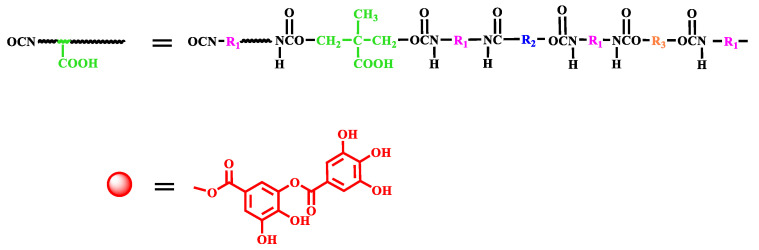
Synthetic procedure of synergistically modified waterborne polyurethane PTWPU.

The synthetic procedure is shown in Scheme 1. In brief, accurately weighed amounts of IPDI (9.99 g), PPG-1000 (14.53 g), PDMS (8% by mass of the total prepolymer, 2.35 g) and DMPA (1.72 g) were added sequentially to a dry three-necked flask with one drop of the catalyst DBTDL, and the reaction was performed under mechanical stirring at 85 °C for 2 h. Subsequently, BDO (0.56 g) was added, and the reaction was continued for 1 h. Next, the TA solution, obtained by dissolving the accurately weighed TA (0.18 g) in an appropriate amount of DMF, was dropped into the flask, and the reaction continued for 3 h at the same temperature. The reaction system was then cooled to room temperature, and the TEA (1.30 g) aqueous solution was added into the three-necked flask for neutralization and emulsification under high-speed stirring for 30 min. Upon termination of the reaction, a synergistically modified waterborne polyurethane emulsion with a solid content of ~30% was obtained, which was designated as PTWPU. It should be noted that the polyurethane control sample without PDMS and TA modification was prepared by the same procedure and named WPU-C.

### 2.3. Preparation of TPTWPU Composite Emulsion and Its Membrane

A certain amount of PTWPU emulsion was placed in a beaker, and then the TiO_2_ nanoparticles, prepared according to our previous report [20], were added (Text S1). The mixture was initially dispersed under magnetic stirring for 5 min and then ultrasonically treated for 5 min until it was homogeneous, thereby obtaining a TPTWPU composite emulsion. Specifically, based on the TiO_2_ dosages of 0.5 wt% and 1.0 wt% relative to the solid content of the PTWPU emulsion, the composite emulsions were named TPTWPU-0.5 and TPTWPU-1.0, respectively.

For preparing the TPTWPU membranes, the corresponding emulsions were cast in PTFE Petri dishes (90 mm × 90 mm), left at room temperature for 12 h to allow the solvent to evaporate naturally, and then transferred to an oven at 50 °C for 24 h. The obtained membrane samples have a thickness of about 0.25 mm. For comparison, the PTWPU and WPU-C membranes were prepared by the same method.

### 2.4. Characterization and Testing

Detailed characterization and testing methods are summarized in the Supplementary Materials.

## 3. Results and Discussion

### 3.1. FTIR and XPS Analysis

**Figure 1:**
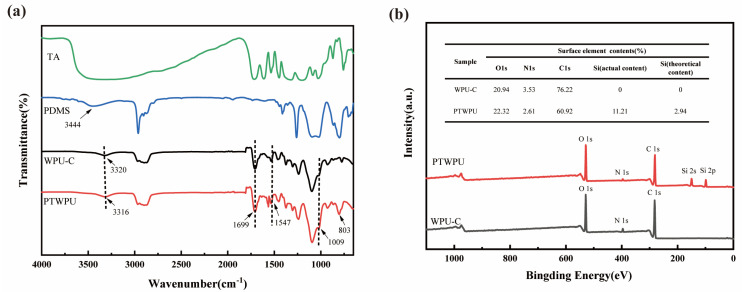
(**a**) FT−IR spectra and (**b**) XPS spectra and elemental analysis (inset) of WPU−C and PTWPU membrane surfaces.

To confirm the successful preparation of waterborne polyurethane synergistically modified with silicone and tannic acid, FT-IR characterization was conducted. FT-IR spectra (Figure 1a) shows that both PTWPU and control WPU-C have characteristic absorption peaks at 1699 cm^−1^ and 1547 cm^−1^, which are attributed to the C=O stretching vibration and the C-N-H bending vibrations (amide II band) respectively, confirming the formation of carbamate groups [29]. As shown in Figure 1a, the absence of absorption bands in the 2260–2280 cm^−1^ region indicates that the NCO groups were completely consumed during the synthesis and emulsification processes. This confirms the absence of residual isocyanate, thereby eliminating toxicity concerns for subsequent applications [30]. In addition, there are peaks at 2942 cm^−1^ and 2855 cm^−1^, corresponding to the stretching vibrations of the -CH_3_ and -CH_2_ groups, respectively [31]. PDMS exhibits a significant O-H stretching vibration peak at approximately 3444 cm^−1^; however, this obvious absorption peak is not observed in PTWPU, indicating that the PDMS soft segments containing terminal hydroxyl groups participated in the chemical reaction, and that the reaction was essentially complete [32]. Compared with the control WPU-C, a new characteristic peak appears at 803 cm^−1^ in the modified PTWPU spectrum, which is attributed to the vibration absorption of the Si-C bond in Si-CH_3_. More importantly, compared with WPU-C, the absorption peak intensity at 1009 cm^−1^ in PTWPU is significantly enhanced, which corresponds to the overlapping region of Si-O-Si and C-O-C stretching vibrations. This intensity enhancement phenomenon indicates the presence of Si-O-Si groups, which further confirms that PDMS has been successfully grafted onto polyurethane molecular chains [33]. In addition, the unmodified WPU-C has an N-H absorption peak at 3320 cm^−1^, and the stretching vibration peak of N-H migrated to 3316 cm^−1^ after the addition of TA, which is attributed to the existence of hydrogen bonding between the phenolic hydroxyl groups in TA and the N-H and C=O groups of the urethanes [34,35]. In addition, a characteristic absorption peak is observed at 1584 cm^−1^, which is attributed to the stretching vibration of the C=C bond in the benzene ring backbone [36]. This directly confirms that TA has participated in the reaction and successfully incorporated into the polyurethane molecular chain, since TA is the only component containing the benzene ring structure used in the synthesis of the modified polyurethane. The appearance and properties of the PTWPU emulsion are detailed in the Supplementary Materials Text S3.

The surface elements of WPU-C and PTWPU membranes were measured by XPS, and their energy spectra and calculated content contents are shown in Figure 1b. The characteristic signals of carbon (C1s), nitrogen (N1s), and oxygen (O1s) appear in the XPS spectra of WPU and PTWPU membranes, which are about 285 eV, 400 eV, and 531 eV, respectively. Obviously, compared with the WPU-C membrane, the PTWPU membrane possesses two different characteristic signals at 100 eV and 150 eV, which correspond to Si 2p and Si 2s, respectively. These results indicate that PDMS has been successfully introduced into the polyurethane molecular chain. Furthermore, the embedded table in Figure 1b shows that the experimental value of the Si element of the PTWPU membrane reached 11.21, which is much higher than the theoretical value of 2.94. This may be due to the low interface energy of flexible PDMS chains, which makes it prone to migrate to the surface during membrane formation, resulting in the enrichment of Si elements, which is beneficial to the surface’s waterproof performance [37].

### 3.2. Microstructure of PTWPU and TPTWPU Composite Membranes

**Figure 2:**
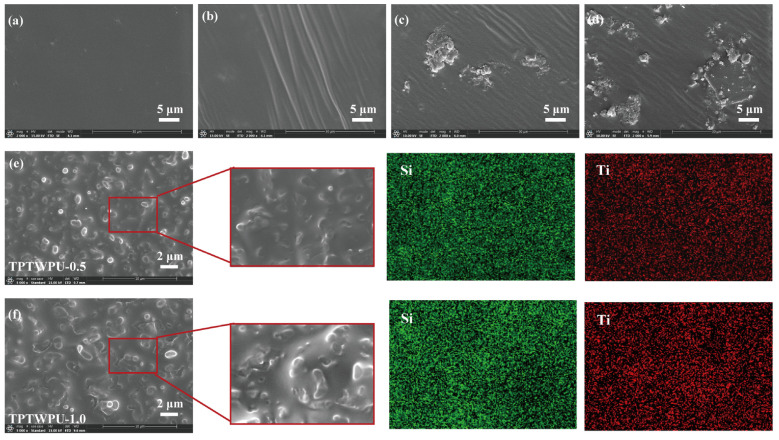
SEM images of the surfaces of (**a**) WPU-C, (**b**) PTWPU, (**c**) TPTWPU-0.5, and (**d**) TPTWPU-1.0 membranes; SEM images and EDS mappings of the cross-sections of (**e**) TPTWPU-0.5 and (**f**) TPTWPU-1.0 membranes.

The microstructure of the PTWPU and TPTWPU composite membranes was characterized by SEM observation. Figure 2a,b show that the surface of the unmodified WPU-C is very smooth, while the surface of the PTWPU membrane modified by PDMS and TA is wrinkled, which is due to the migration and enrichment of hydrophobic and flexible Si-O-Si chain segments to the membrane surface, thus increasing the roughness of the membrane surface. Figure 2c,d show that the surfaces of the composite membranes TPTWPU-0.5 and TPTWPU-1.0 are also wrinkled, and a few nanoparticles are partially exposed on the surface in the form of agglomerates. It can be speculated that the enrichment of hydrophobic siloxane components on the surface and the increase in surface micro-roughness endowed by nanoparticles will contribute to enhancing the waterproof property of the membrane. In addition, to investigate the distribution of TiO_2_ nanoparticles within the membrane, the cross-sectional morphology and elemental distribution of the composite membrane were observed by SEM and EDS mapping. Figure 2e,f show that the distribution of Ti elements is uniform, indicating that nanoparticles are well dispersed inside the composite membrane. This is beneficial to the construction of hydrophilic channels in the membrane and the enhancement of moisture permeability. However, compared with TPTWPU-0.5, the nanoparticles in the TPTWPU-1.0 membrane are slightly aggregated. In addition, it can be found that the distribution of Si elements is also uniform, indicating that the polydimethylsiloxane component is chemically bound to the polyurethane molecular chain. This further proves the realization of the design idea of silicone-modified polyurethane.

### 3.3. Mechanical Properties and Thermal Stability of PTWPU and TPTWPU Composite Membranes

**Figure 3:**
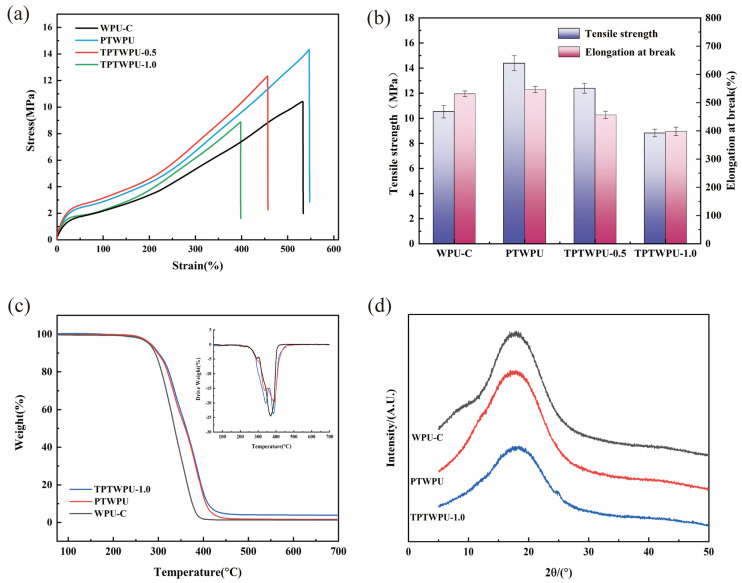
(**a**) Stress−strain curves, (**b**) mechanical data, (**c**) TG and DTG (inset) curves, and (**d**) XRD patterns of different membranes.

Figure 3a presents the stress–strain curves of PTWPU and TPTWPU membranes, and the corresponding mechanical data are shown in Figure 3b. Compared with WPU-C membranes, PTWPU membranes modified by PDMS and TA show increased tensile strength and elongation at break. This improvement can be due, on the one hand, to the flexible nonpolar siloxane chain playing the role of plasticizer, while on the other hand, abundant phenolic hydroxyl groups on TA molecules react with polyurethane backbone chains to form phenol–urethane bonds, leading to crosslinking, and this strong interaction strengthens the intermolecular forces. As a consequence, both tensile strength and elongation at break are improved [38]. In contrast, the introduction of TiO_2_ nanoparticles reduced the mechanical properties of TPTWPU membranes. Moreover, the more nanoparticles are added, the greater the reduction is. This may be contributed to by the introduction of inorganic nanoparticles into the polyurethane matrix through physical blending, which leads to phase separation and reduces the interaction between polymer molecular chains. Additionally, the aggregation of nanoparticles in the membrane may further compromise the tensile strength and elongation at break [39].

Figure 3c presents the thermogravimetric (TG) curves of different sample membranes. All of these curves exhibit three distinct stages of weight loss. The first stage, occurring between 100 °C and 270 °C, is primarily attributed to the evaporation of adsorbed water and the decomposition of some oligomers. The second stage, from 280 °C to 350 °C, corresponds mainly to the thermal decomposition of the hard segments of the waterborne polyurethane, degrading into isocyanates and polyols. The third stage, between 360 °C and 420 °C, involves the thermal decomposition of the soft segments and part of the polysiloxane chain segments [40]. From the TG curve of PTWPU membrane, it can be seen that the thermal decomposition temperature is increased after being modified by PDMS and TA, indicating that the thermal stability of the PTWPU membrane is improved. This may be explained by the following two reasons: (1) The Si-O bond in PDMS has a relatively high bond energy and is not easy to decompose, which enhances the thermal stability of the polyurethane membrane. When faced with high temperatures, PDMS tends to decompose to form inorganic silica particles, which impede further thermal degradation of polyurethane molecular chains. (2) TA increases the degree of crosslinking and the density of the membrane, which will hinder full contact between oxygen and the polyurethane matrix and prolong the volatilization of decomposition products, thus enhancing the thermal decomposition temperature [38]. It is worth mentioning that after incorporating TiO_2_ nanoparticles, although no significant increase in thermal decomposition temperature was observed, the residual decomposition amount did increase. This indicates that the introduction of TiO_2_ enhances the thermal stability of polyurethane membrane.

Figure 3d presents the XRD patterns of various sample membranes. A broad characteristic diffraction peak near 19.8°, attributed to the soft segment, is observed in all sample membranes, confirming the amorphous nature of the synthesized polyurethane. Moreover, the intensity of this broad peak successively decreases in the order of WPU-C, PTWPU, and TPTWPU. This phenomenon arises primarily from the grafting of PDMS onto the side chain of polyurethane molecules, which increases steric hindrance, weakens the intermolecular forces, and disrupts the structural regularity of the polyurethane, thereby lowering its crystallinity [37]. Furthermore, for the TPTWPU membrane, the incorporation of nanoparticles restricts segmental mobility and intermolecular stacking in the polyurethane matrix, leading to a further decrease in ordered crystalline domains. It is worth noting that the characteristic peaks of TiO_2_ nanoparticles are not seen in the XRD pattern of the TPTWPU membrane, which may be due to its low dosage.

### 3.4. Waterproofing and Moisture Permeability of PTWPU and TPTWPU Composite Membranes

**Figure 4:**
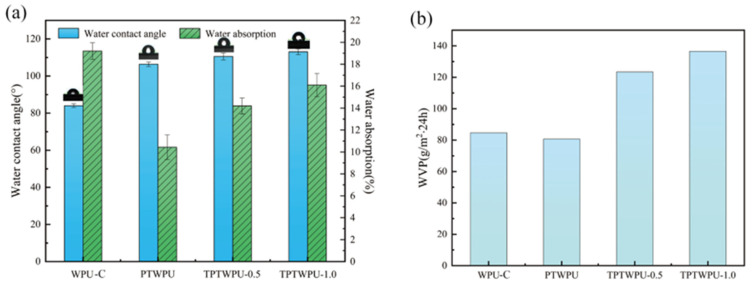
(**a**) Water contact angle and water absorption of different sample membranes. (**b**) Moisture permeability of different sample membranes.

Figure 4a demonstrates the water contact angle (WCA) and water absorption of the PTWPU membrane and the TPTWPU composite membrane compounded with TiO_2_ nanoparticles. It can be seen that the WCA of the unmodified WPU-C membrane is only 82.3°, and the water absorption is nearly 20%. After modification with PDMS and TA, the WCA of PTWPU membrane increased significantly, reaching 106.8°, and the water absorption decreased to about 10%. This is consistent with the results of XPS analysis of surface silicon content and SEM observation of surface microstructure. Furthermore, after adding TiO_2_ nanoparticles, compared with PTWPU, the WCA of the TPTWPU membrane increased slightly, and the water absorption rate also increased. Moreover, they further increased with the increase in TiO_2_ content, after which the WCA of TPTWPU-1.0 reached 115.3° and its water absorption reached 16.1%. This is because TiO_2_ nanoparticles are exposed on the surface of the membrane, as revealed by SEM observation (Figure 2), which increases the roughness of the surface and enhances its hydrophobicity [41]. In addition, the surface of TiO_2_ nanoparticles contains hydroxyl groups and is hydrophilic, which leads to an increase in hydrophilicity inside the membrane, so the water absorption rate is increased. Therefore, it can be inferred that for the TPTWPU membrane, through the introduction of hydrophilic TiO_2_ nanoparticles, fine pores are formed between inorganic particles and organic polyurethane molecular chains, and a good hydrophilic channel for water vapor transmission via an adsorption–diffusion mechanism is constructed inside the membrane, which is beneficial to the mass transfer and diffusion of moisture in the membrane and will improve the moisture permeability. On the other hand, the low-surface-energy PDMS segments migrate to the membrane–air interface to form an effective barrier against liquid water, as well as the surface micro-roughness formed by nanoparticles, so the contact angle of the membrane surface remains even further increased, which indicates that good waterproof performance can be obtained at the same time.

Subsequently, the moisture permeability of WPU-C, PTWPU, TPTWPU-0.5, and TPTWPU-1.0 composite membranes is detected and shown in Figure 4b. Obviously, the water vapor permeability (WVP), that is, the moisture permeability of unmodified WPU-C and PTWPU membranes, is poor. For the PTWPU membrane, this is because the introduction of PDMS increases the aggregation of silicon elements on the surface and enhances the water repellency, while the crosslinking of TA makes the internal structure of the membrane more compact, leading to a decrease in moisture permeability. As for TPTWPU membranes, the WVP is greatly improved after the incorporation of TiO_2_ nanoparticles. Specifically, compared with the PTWPU membrane, the WVP of TPTWPU-0.5 increased by approximately 45.8%, while that of TPTWPU-1.0 increased by approximately 61.2%. These results may stem from the enlargement of pores within the polyurethane matrix by TiO_2_ nanoparticles and the hydrophilicity caused by the rich hydrophilic hydroxyl groups on its surface. Therefore, water vapor molecules can effectively diffuse to the hydrophobic outside of the membrane along these hydrophilic groups and hydrophilic pathways, and thus the TPTWPU membrane exhibits excellent waterproofing and moisture permeability. The successful construction of this kind of double-surface characteristic membrane, that is, a composite membrane combining a hydrophobic surface with a hydrophilic interior, perfectly integrates two traditional competitive properties, providing an effective method for the development of waterproof and moisture-permeable membranes.

### 3.5. Photocatalytic Performance and Self-Cleaning Property of TPTWPU Composite Membranes

**Figure 5:**
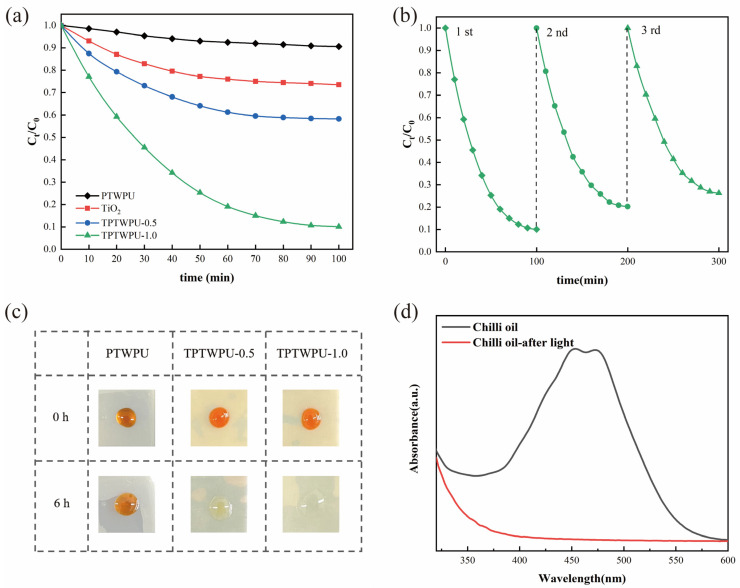
(**a**) Degradation curves of samples in methylene blue solution under visible light conditions. (**b**) Cyclic stability test of TPTWPU-1.0 membrane. (**c**) Self-cleaning performance of TPTWPU composite membrane. (**d**) UV-Vis spectra of chili oil before and after light exposure.

To investigate whether TPTWPU membranes possess self-cleaning abilities, we first tested their photocatalytic performance. Figure 5a presents the photocatalytic degradation curves of various samples under visible light irradiation, using methylene blue as a simulated pollutant. Two control samples were designed to perform experiments: one used the PTWPU membrane (without TiO_2_), and the other used pure TiO_2_ photocatalyst powder with the same amount as that contained in TPTWPU-1.0. Clearly, both controls exhibited relatively low degradation efficiency. In contrast, TPTWPU composite membranes demonstrated significantly enhanced photocatalytic performance; especially, the TPTWPU-1.0 composite membrane achieved a degradation rate of over 90%. These results can be interpreted as follows: The PTWPU membrane itself lacks photocatalytic activity, while TiO_2_ particles are prone to agglomerate and some active sites may be shielded, which is not enough to effectively degrade dye solution. For the TPTWPU composite membrane, PTWPU, as the carrier of TiO_2_ photocatalysts, can disperse it well and effectively prevent the issues of agglomeration tendency and insufficient contact. Moreover, TA in the TPTWPU membrane can form a synergistic effect with TiO_2_ photocatalysts; that is, as an electron trap and carrier, TA can effectively suppress the electron–hole recombination of photocatalysts, prolong the lifetime of electron–hole pairs, and thereby enhance the overall catalytic activity [42]. In addition, Figure 5b shows the results of the recycling test of the TPTWPU-1.0 membrane. It can be seen that the degradation efficiency decreased to some extent after three cycles, but it was still above 75%. The reason may be that the TiO_2_ photocatalyst will be partially shed during photocatalytic degradation, but there may be complexation of unreacted phenolic hydroxyl groups of TA with metal ions in the polyurethane matrix [43], which can also prevent the agglomeration of nanoparticles, thus making their photocatalytic performance effective and stable [44].

After confirming the photocatalytic activity of TPTWPU, the self-cleaning experiment was carried out, using chili oil as a model contaminant. A droplet of chili oil was placed on composite membranes with different TiO_2_ loadings. ThenPTWPU membrane without TiO_2_ nanoparticles served as the control. All the samples were then exposed to natural sunlight for 6 h. Figure 5c shows that after natural light irradiation, the oil stain on the surface of TPTWPU-0.5 is almost invisible, and the color on the surface of the TPTWPU-1.0 membrane has been completely removed, while the oil stain on the surface of the PTWPU membrane, used as a control, has no obvious change, indicating that TPTWPU composite membranes have a self-cleaning effect on oily stains. In order to further illustrate this self-cleaning effect, the UV-Vis absorption spectra of chili oil before and after irradiation were tested. Figure 5d shows that the characteristic absorption peak in the range of 450–480 nm [45], which belongs to the conjugated polyene chain of carotenoids that produce the color of chili oil, has disappeared after illumination, indicating that it has been fractured. This is attributed to the fact that TiO_2_ nanoparticles destroy the conjugated double bonds by generating hydroxyl radicals (-OH) and holes (h^+^) [46], which macroscopically showed that chili oil changed from reddish-yellow to colorless and transparent.

### 3.6. Self-Healing Performance of TPTWPU Composite Membrane

**Figure 6:**
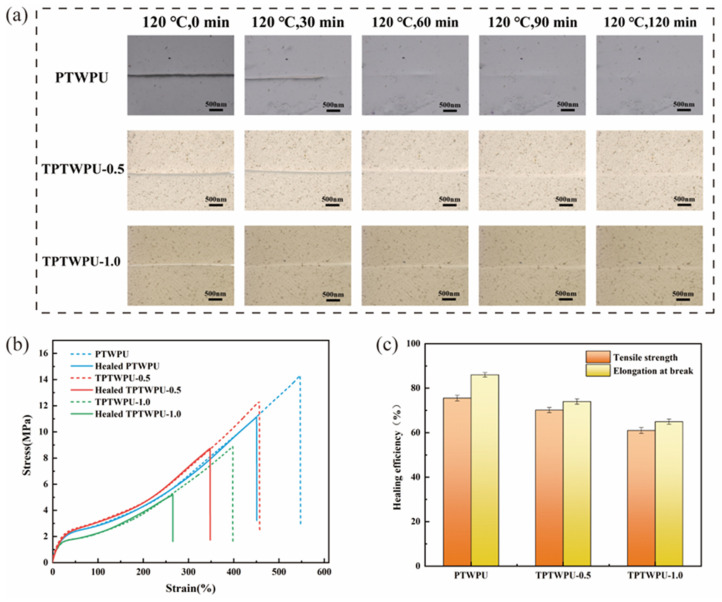
(**a**) Self-healing test of PTWPU and TPTWPU membranes; (**b**) stress–strain curves and (**c**) self-healing efficiency of PTWPU and TPTWPU membranes before and after self-healing test.

It is reported that after TA is introduced into waterborne polyurethane as a crosslinking agent, dual dynamic crosslinking networks (phenolic carbamate covalent crosslinking network and hydrogen-bonding crosslinking network) can be constructed, which endow the material with self-healing performance and allow the bonds to be broken and recombined at 120 °C [25]. To verify this property, the as-prepared PTWPU and TPTWPU membrane samples were subjected to fracture, and then the repair process was observed at 120 °C, and the results are shown in Figure 6a. It can be seen that the self-healing effect of the PTWPU membrane is the most outstanding, and the incision has been basically reattached at 60 min, and almost no trace can be seen at 120 min. However, after adding nanoparticles, the self-healing effect of the TPTWPU-0.5 and TPTWPU-1.0 membranes both decreased, but they all healed completely within 120 min. Figure 6b,c show the stress–strain curves and the corresponding self-healing efficiency before and after the self-healing test of the membranes, respectively. It can be seen that the recovery rates of tensile strength and elongation at break of the PTWPU membrane are around 80%, which is contributed to by the high temperature triggering the reversible transformation under the joint action of a phenolic carbamate network and a hydrogen-bonding crosslinking network to get a better self-healing effect in the incision. However, the self-healing efficiencies of TPTWPU-0.5 and TPTWPU-1.0 membranes decreased but fortunately remained above 60%. This phenomenon may be due to the isolation effect of the incorporated TiO_2_ nanoparticles, which weakens the interaction between polyurethane molecular chains and reduces their mobility within the polymer matrix.

## 4. Conclusions

In summary, this study successfully synthesized waterborne polyurethane (PTWPU) modified by polydimethylsiloxane (PDMS) and tannic acid (TA) using PDMS as a hydrophobic modifier and TA as a crosslinker. This synergistic modification strategy effectively enhances the surface hydrophobicity of the membrane, and at the same time, the phenolic carbamate network formed exhibits favorable dynamic characteristics to obtain self-healing performance. Subsequently, TPTWPU composites are prepared by incorporating TiO_2_ photocatalyst nanoparticles. The hydrophobicity is further improved by constructing surface micro-roughness, and hydrophilic channels are built inside the membrane to facilitate the mass transfer and diffusion of moisture and achieve moisture permeability. Meanwhile, the TA-promoted photocatalytic activity is imparted to realize self-cleaning performance. The results show that the representative TPTWPU-1.0 (PTWPU with 1.0 wt% TiO_2_) waterproof and moisture-permeable membrane possesses a WCA of 115.3° and a moisture permeability of 135.0 g/(m^2^·24 h) (representing an increment of 61.2% relative to the unmodified membrane). Its degradation rate of methylene blue is over 90% under visible light, and it shows an effective self-cleaning capability for chili oil under natural light. Meanwhile, it shows excellent self-healing performance, with cuts fully recovering after heating at 120 °C for 2 h, and a healing efficiency exceeding 80%. This work offers a feasible strategy for designing and constructing multifunctional waterproof and moisture permeable membranes, with great potential application prospects in multifunctional coatings for leather and textile finishing.

## Data Availability

The original contributions presented in this study are included in the article. Further inquiries can be directed to the corresponding author.

## Supplementary Materials

The following supporting information can be downloaded at https://www.mdpi.com/article/10.3390/polym18070881/s1, Text S1. The preparation of TiO_2_ nanoparticles; Text S2. Characterization and Testing; Text S3. Appearance, particle size and molecular weight of the PTWPU emulsion; Figure S1. (a) Appearance, (b) particle size and (c) molecular weight distribution of the PTWPU emulsion.
