# Supplementary material for: Waterproof and Breathable Polyurethane Membranes with Self-Healing and Self-Cleaning Properties: Synergistic Enhancement by Polydimethylsiloxane and Phenolic Carbamate Network and Photocatalytic Effect
Source: Polymers (Basel). 2026 Apr 3;18(7):881. doi: 10.3390/polym18070881 (PMC13075008; doi:10.3390/polym18070881)
Supplement: Supplementary file 1 [file polymers-18-00881-s001.zip › polymers-4215226-supplementary.pdf]

Supplementary Materials

Waterproof and Breathable Polyurethane Membranes with Self-healing and Self-cleaning Properties: Synergistic Enhancement by Polydimethylsiloxane and Phenolic Carbamate Network and Photocatalytic Effect

Yuqing He [1,2] , Xiaohan Yang [1,2], Fufen Li [1,2], Xiudan Tao [1,2], Chenhui Liu [1,2], Zhengjun Li [1,2,*]

[1] Key Laboratory of Leather Chemistry and Engineering of Ministry of Education, Sichuan University, Chengdu 610065, China

[2] College of Biomass Science and Engineering, Sichuan University, Chengdu 610065, China

[*] Correspondence: lizhengjun@scu.edu.cn (Zhengjun Li)

Text S1. The preparation of TiO_2 nanoparticles

Anhydrous ethanol (15 mL), deionized water (3 mL), and concentrated nitric acid (0.02 mL) were sequentially added to a beaker. Subsequently, a mixture containing 13.6 g of titanium tetrabutoxide (TBT), 26 mL of anhydrous ethanol, and 0.7 mL of acetone was added dropwise under continuous stirring for 2 h to obtain a transparent sol. The sol was then allowed to age at room temperature for 24 h, resulting in a pale yellow gel. The gel was spread evenly in a Petri dish, dried at 80°C for 24 h, and then ground and sieved. Finally, the powder was calcined in a muffle furnace at 500°C for 2 h to yield titanium dioxide nanoparticles.

Text S2. Characterization and Testing

The solid content of the PTWPU dispersion was determined by gravimetric analysis. Approximately 2 g of the emulsion sample (m_1) was placed in an aluminum dish and dried in an oven at 105°C until constant weight (m_2). The solid content (S) was calculated using the following Equation (S1):

$$S=\frac{m_2}{m_1}\times 100\% \qquad \text{(S1)}$$

The overall yield (Y) of the TPWPU was calculated based on the solid content, as the

product is obtained in the form of an aqueous dispersion. The total solid mass obtained was determined by multiplying the total mass of the final dispersion (M_d) by its solid content (S). The yield was then calculated according to the following Equation (S2):

$$Y=\frac{M_d \times S}{M_t}\times 100\% \quad \text{(S2)}$$

Where M_t is the total mass of all non-volatile feedstocks.

The particle size of the emulsion was measured using dynamic light scattering (DLS) technology (Nano-ZEN3600, UK).The molecular weight of the emulsion was determined using gel permeation chromatography (GPC) (Agilent 1260 Infinity II, USA).The Fourier transform infrared (FT-IR) spectra of the thin membranes were determined using a Nicolet ATR-FTIR spectrometer in the wave number range of 4000 to 650 cm^{-1}. The morphology of the membranes was observed using a scanning electron microscope (SEM) (Apreo S Hi Vac, USA). The crystallinity of the membranes was evaluated using an X-ray diffractometer (XRD) (The Netherlands, DY129) with a scanning speed of 4° /min and a 2θ angle in the range of 5° to 60°. The optical properties of the samples were evaluated using an ultraviolet-visible spectrophotometer (UV-Vis) (UV3900H, Switzerland) with a wavelength range of 200-800 nm and a scanning step of 2 nm. The tensile strength and elongation at break of the membranes were measured using a universal electronic tensile machine (UTM6203, China) at a strain rate of 100 mm/min.

The water resistance of the membranes were evaluated by water contact angle (WCA) and water absorption. The WCA of the membranes was tested using a HKCA-40 contact angle analyzer (Beijing, China). For the water absorption test, the mass of dry membrane was measured as W_1, and then the membrane was immersed in deionized water at 25°C for 24 h. After that, the water on the surface of sample was wiped off, and the mass of the sample was recorded as W_2. The water absorption W (%) was calculated by the following Equation (S3).

$$W=\frac{W_2-W_1}{W_1}\times 100\% \quad \text{(S3)}$$

Reference to GB/T 1037-2021 , the moisture permeability of the membranes was tested using a YG(B)751DG constant temperature and humidity chamber (Wenzhou, China). The water vapor transmission rate of the membranes was determined based on the cup weight gain method and was calculated by Equation (S4).

$$WVP = \frac{24\times(m_1-m_2)}{S\cdot t} \quad (S4)$$

Where WVP is the water vapor permeability (g/(m^2·24h)), S is the sample area (m$^{2)}$, t is the interval time between two measurements (h), and m_0 and m_1 are the initial mass and the mass at the time t (g), respectively.

The photocatalytic activity of the membranes were evaluated by monitoring their degradation efficiency of methylene blue (MB) in aqueous solution under simulated sunlight irradiation. Prior to irradiation, membrane samples (2.0×2.0 cm) were immersed in a 10 mg/L MB solution in darkness for 30 min to establish adsorption-desorption equilibrium. Subsequently, photocatalytic degradation was conducted under illumination from a 150 W xenon lamp simulating visible light. Solution absorbance was measured via ultraviolet-visible spectrophotometry, with MB concentration changes recorded every 10 min.

The self-cleaning performance of the membranes were evaluated by dropping 50 μL of chili oil on their surface, and then observing the color change and residual condition of the oil stains under visible light.

For the self-healing test, the membrane was first cut into 50 mm×4 mm×0.3 mm dumbbell-type sample strips, and the strips were cut off in the middle with a clean razor blade, and then self-healed for a period of time in a 120ºC constant-temperature oven, and the micromorphology of the membrane at the incision was observed with a stereo microscope every half an hour, and then the heating was stopped when no scratches could be seen on the surface of the membrane. The mechanical properties were tested after the strips were stored for 24 h at room temperature[1], and the recovery rate of the sample strip was calculated according to Equation (S5).

$$\eta=\frac{\sigma_1}{\sigma_2}\times100\% \quad (S5)$$

Where η is the recovery rate, σ_1 and σ_2 are the mechanical property parameters of the membrane after and before self-healing, respectively.

Text S3. Appearance, particle size and molecular weight of the PTWPU emulsion

As shown in the figure S1, the PTWPU emulsion appears as a pale yellow, opaque liquid with a mean particle size of approximately 102 nm. The calculated yield of PTWPU is 94.6%. Additionally, GPC analysis reveals that the average weight molecular weight

(Mw) of the PTWPU polyurethane is 24,527 g/mol.

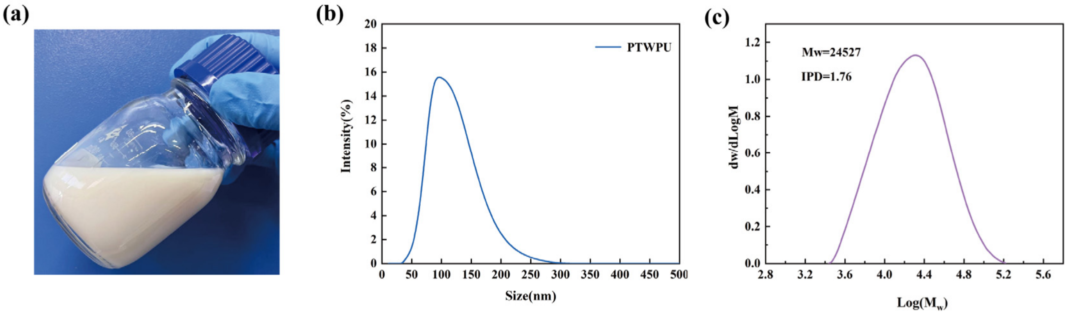

Figure S1. (a) Appearance, (b) particle size and (c) molecular weight distribution of the PTWPU emulsion

References

1.Liu, Y.; Zhang, Z.T.; Fan, W.W.; Yang, K.; Li, Z. Preparation of renewable gallic acid-based self-healing waterborne polyurethane with dynamic phenol-carbamate network: Toward superior mechanical properties and shape memory function. J. Mater. Sci. 2022, 57, 5679–5696.
